# Survival in Norwegian BRCA1 mutation carriers with breast cancer

**DOI:** 10.1186/1897-4287-7-7

**Published:** 2009-04-14

**Authors:** Anne Irene Hagen, Steinar Tretli, Lovise Mæhle, Jaran Apold, Nina Vedå, Pål Møller

**Affiliations:** 1 Department of Laboratory Medicine, Children's and Women's Health, Faculty of Medicine, Norwegian University of Science and Technology, Trondheim, N-7006 Trondheim, Norway; 2 Department of Surgery, St. Olav's Hospital, Trondheim University Hospital, N-7006 Trondheim, Norway; 3 The Norwegian Cancer Registry, N-0310 Oslo, Norway; 4 Department of Public Health and General Practice, Norwegian University of Science and Technology, Trondheim, Norway; 5 Section for Inherited Cancer, Department of Medical Genetics, Rikshospitalet-Radiumhospitalet Medical Center, N-0310 Oslo, Norway; 6 Institute of clinical medicine, University of Bergen, N-5021 Bergen, Norway; 7 Centre of Medical Genetics and Molecular Medicine, Haukeland University Hospital, N-5021 Bergen, Norway

## Abstract

Several studies of survival in women with *BRCA1 *mutations have shown either reduced survival or no difference compared to controls. Programmes for early detection and treatment of inherited breast cancer, have failed to demonstrate a significant improvement in survival in *BRCA1 *mutation carriers.

One hundred and sixty-seven women with disease-associated germline *BRCA1 *mutations and breast cancer from 1980 to 2001 were identified. Tumour characteristics, treatment given and survival were recorded. A control group comprising three hundred and four women matched for age, time of diagnosis and stage were used to compare survival.

*BRCA1 *mutation carriers were found to have a poorer prognosis, which could be explained by neither the mode of surgical treatment nor the use of adjuvant chemotherapy. *BRCA1 *mutation carriers with node negative breast cancer had worse overall survival than controls.

Our findings confirm the serious prognosis of *BRCA1*-associated breast cancer even when diagnosed at an early stage, and that type of treatment does not influence prognosis.

## Background

*BRCA1*-associated breast cancers differ from sporadic breast cancer with regard to prognostic markers. *BRCA*-associated breast cancers are usually high grade, hormone receptor- and HER-2 negative and often express topoisomerase IIa [1-4]. Studies of survival for *BRCA1*-associated breast cancer based on mutation analysis have come to varying conclusions: None has reported better survival rate, many have reported no differences [5-7]. Two case-control studies of early stage breast cancer [8,9] and a study with prospectively ascertained cancers [10] failed to show any survival disadvantage. However, a number of studies have reported worse prognosis [11-19].

A study on the effects of interventions to prevent inherited breast cancer in prospectively ascertained breast cancers in screened women at family history clinics, indicated that *BRCA1 *mutation carriers have less effect of early diagnosis and treatment compared to other groups [20]. If this is so, improved early diagnosis by means of annual breast magnetic resonance imaging (MRI) in addition to annual mammography, which has been shown to improve early diagnosis [21], may not be accompanied by an improved survival rate.

In the present study, we compared survival in *BRCA1 *mutation carriers with mode of treatment. Also, we compared survival in *BRCA1 *mutation carriers with a control group with sporadic breast cancer matched for age, stage and time of diagnosis.

## Materials and methods

All women found to be *BRCA1 *mutation carriers and diagnosed with breast cancer in the years 1980 to 2001 were identified in the archives of Section for Inherited Cancer, Department of Medical Genetics, Rikshospitalet-Radiumhospitalet Medical Center, Oslo and Centre of Medical Genetics and Molecular Medicine, Haukeland University Hospital, Bergen. These two registers contain about 95% of known *BRCA1 *mutation carriers in Norway. All participating patients had given their consent during genetic counselling. Permission from the Norwegian Data Inspectorate had been given to gather the data from these two archives and analyze them for scientific purposes. Index patients and obligate carriers are included.

From 1980 treatment of breast cancer in Norway was influenced by the guidelines from the Norwegian Breast Cancer Group (NBCG) and was rather homogenous in the whole country. For this reason we chose not to go further back than 1980. Also, stage at diagnosis was considered less accurate before 1980.

The control group was found in the Norwegian Cancer Registry matched for age, (+/- one year), time of diagnosis (+/- one year) and stage. It was not always possible to find two controls within the matching criteria. One hundred and forty-five cases have two controls, 14 cases have one control and eight have none.

For mutation carriers data on surgical treatment, histopathological findings, treatment given and the course of the disease were extracted from the archives of the departments of genetics. Any missing information was retrieved from hospital records. Due to limited resources and the retrospective collection of data, no histopathological review was done, all information was based on the original report given at the time of diagnosis. Interpretation of immunohistochemical staining for estrogen receptor (ER) varied from centre to centre. In most cases, histopathological grading, according to Bloom and Richardson [22], modified by Elston and Ellis [23], was done. However, grading had not been done in fifty-three (31.7%) cases.

ER status and histopathological grading were not available for the controls. Because presence of a *BRCA1 *mutation is strongly associated with absence of ER receptor and high grade, selection of controls by these parameters would have implied an element of informative censoring – selecting controls by parameters associated with the selection criterion for the cases.

Survival of mutation carriers was calculated inside the electronical medical files. Survival in controls was calculated inside the electronical files of the Cancer Registry of Norway. To compare survival from the different series, pseudonymized data were exported and no research registry was erected. All data collected were derived from standard health care procedures, no patient was examined, no sample was obtained, and no specimen was re-examined for this study.

### Statistics

The survival analyses were performed using the Kaplan-Meier model in SPSS version 14.0 (SPSS Inc., Chicago, IL, USA). Death was scored as event. For calculation of contralateral cancer incidence, patients were censored at contralateral prophylactic mastectomy and contralateral reduction operation. Patients were censored at oophorectomy when considering ovarian cancer.

## Results

A number of findings in the mutation carriers are detailed in Table 1: Mean age at diagnosis was 44.4 years, median age 43.7, minimum age 27, maximum age 73. Of the 167 cases, 104 (62%) had stage 1 (T1-2N0) disease. They had predominantly ductal cancers (82%). Fifty per cent and 43% had tumour size T1 and T2, respectively, 49% were histopathological grade III, 52% were ER negative, and 61% were without nodal spread. The incidence of local recurrence among carriers operated with breast-conserving therapy (BCT) was 8/40 (20%). Median time to recurrence was 85 months, minimum 29, and maximum 122 months. Bilateral prophylactic salpingo-oophorectomy (BPSO) had been performed in 104/167 (62.3%). Occult ovarian cancer was found in 8/104 (8%) of those who had BPSO. Thirty-four (34/167 (20.4%)) had an oophorectomy due to suspicion of malignancy. Details of the control population are given in table 2.

**Table 1:** Characteristics of the 167 BRCA1 mutation carriers

		no(%)
**Age**	<30	6(3.6)
	30–39	47(28.1)
	40–49	70(41.9)
	50–59	33(19.8)
	60–69	7(4.2)
	>/= 70	4(2.4)
		
**Type of operation**	Mastectomy	125(74.9)
	BCT	40(24.0)
	Ablatio simplex	1(0.6)
	Halsteds	1(0.6)
		
**Stage**	1(T1-2N0)	104(62.3)
	2(T1-2N1)	54(32.3)
	3(T1-2N2-3, T3-4N0-3)	9(5.4)
		
**Size**	T1(tumour ≤2 cm)	83(49.7)
	T2(tumour >2 cm, ≤5 cm	71(42.5)
	T3(tumour >5 cm)	6(3.6)
	T4(Infiltration in skin or muscle, inflammatory)	3(1.8)
	Unknown	4(2.4)
		
**Grade**	I	2(1.2)
	II	21(12.6)
	III	82(49.1)
	Unknown, incl. DCIS, LCIS	62(37.1)
		
**Nodal status**	Negative	101(60.5)
	Positive	66(39.5)
		
**ER status**	Negative	86(51.5)
	Positive	45(26.9)
	Unknown	36(21.6)
		
**Type**	Ductal	137(82.0)
	Medullary	11(6.6)
	Lobular	5(3.0)
	DCIS	6(3.6)
	LCIS	3(1.8)
	Anaplastic	1(0.6)
	Tubular	1(0.6)
	Mucinous	1(0.6)
	Mixed ductal/lobular	1(0.6)
	Mixed medullary/ductal	1(0.6)
		
**Chemotherapy**	Yes	93(55.7)
	No	74(44.3)
		
**Contralateral cancer**	Yes	42(25.1)
	No	110(65.9)
	Prophylactic mastectomy contralat	13(7.8)
	Reduction contralat	2(1.2)

**Table 2:** Age and stage for the 304 controls

		no(%)
**Age**	<30	11(3.6)
	30–39	81(26.6)
	40–49	134(44.2
	50–59	56(18.4)
	60–69	16(5.3)
	>/= 70	6(2.0)
		
**Stage**	1(T1-2N0)	204(67.1)
	2(T1-2N1)	91(29.9)
	3(T1-2N2-3, T3-4N0-3)	9(3.0)

**Figure 1:**
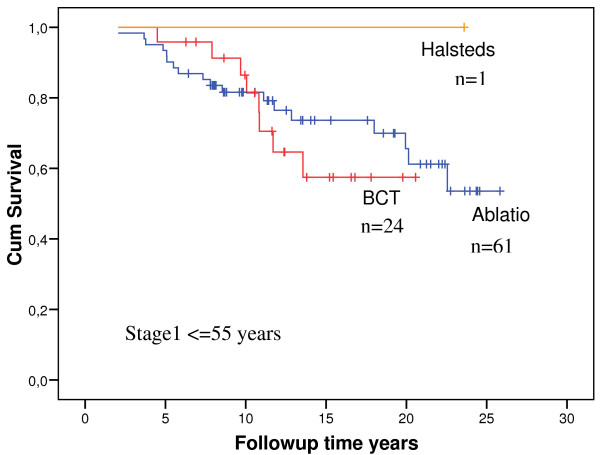
**Overall survival in cases stage 1, age ≤55 years, stratified on type of operation**. Initial numbers in groups are indicated in the figure. Halsteds operation orange line. Breastconserving operation: red line. Mastectomy: blue line.

**Figure 2:**
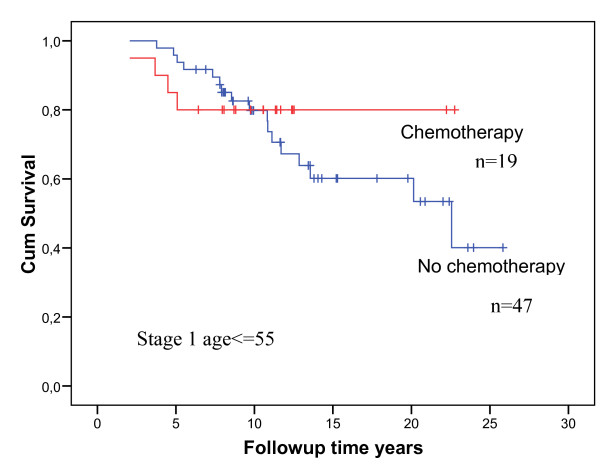
**Overall survival in cases stage 1, stratified on chemotherapy or no chemotherapy (removal of those who had chemotherapy on days 0 and 7)**. Initial numbers in groups are indicated in the figure. No chemotherapy given: Red line. Chemotherapy given: Blue line.

No association with survival for mastectomy compared to BCT was found in survival analyses of all cases or, indeed, when stage 1 was analyzed separately. Figure 1 illustrates the latter in premenopausal cases. Likewise, there was no obvious difference between survival of those having received and those not having received chemotherapy, neither in the total study population nor when stage 1 was analyzed separately (Figure 2). Contralateral cancer was seen in 42/154 (27%). Thirteen had previously undergone contralateral prophylactic mastectomy.

**Figure 3:**
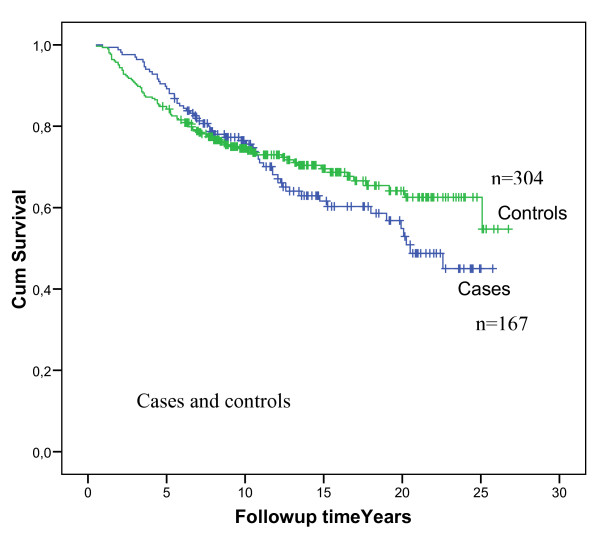
**Overall survival in cases and controls**. Initial numbers in groups are indicated in the figure. Controls: Green line. Cases: Blue line.

**Figure 4:**
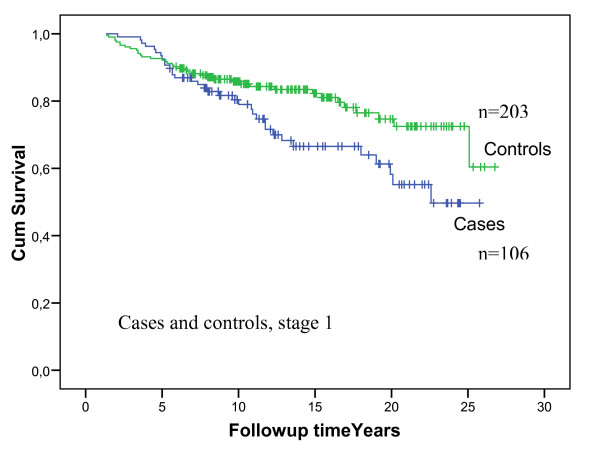
**Overall survival in stage 1 for cases and controls**. Initial numbers in groups are indicated in the figure. Controls: Green line. Cases: Blue line.

In the control group the mean age at diagnosis was 44.9 years. Median follow-up time for carriers and controls together was 10.2 years. Survival analyses of cases compared to controls showed better survival of the cases initially, but later the opposite (Figure 3). A closer look at specific cause of death showed that deaths from ovarian cancer may have contributed to the shape of the survival curve for the carriers. Considering survival of cases compared to controls for stage 1 only, the initial better survival among cases gradually disappeared, and the curves seemed to diverge with a poorer survival in the cases (Figure 4). Should significance testing be applied to such curves, the *p *value would be 0.02.

## Discussion

The results of this study confirm that early diagnosis and treatment did not imply a general favourable prognosis in the *BRCA1 *mutation carriers. This is compatible with the prospective series of cancer cases with early diagnosis. [24]. On the other hand, in the prospective series the prognosis of other groups of breast cancers was favourable. It is possible that some forms of familial or inherited breast cancers are biologically different from both the cases and controls in the current study. Furthermore, early diagnosis and treatment may confer a favourable prognosis in the other groups, but not in *BRCA1 *mutation carriers.

The majority of cases was premenopausal and had stage 1 disease. The results did not indicate that mastectomy was superior to breast conserving surgery with respect to survival, neither in the total group nor in the stage 1 cancers when considered separately. Similarly, there was no obvious effect of chemotherapy. Removing the cases that received initial short-time chemotherapy from the series had no effect on the results (data not shown). However, before concluding that chemotherapy does not improve survival in *BRCA1 *mutation carriers, it should be noted that this statement refers to a study on effect of standard adjuvant chemotherapy as given some years ago. The results may not have bearings on recent discussions on the effect of cisplatin [25,26]. The conclusions were, however, that none of the treatment options employed was associated with, or could explain, the serious prognosis in the *BRCA1 *mutation carriers even in early stage disease at time of diagnosis.

The first impression of the survival of cases compared to controls was confusing, due to the initial better survival in the *BRCA1 *mutation carriers and the later worse survival resulting in overlapping curves. If our findings are representative, they may explain some of the contradictions in the literature: Survival compared to controls may differ depending on the number of years of follow-up. We see a number of factors that may possibly contribute to the observed results. Mutation carrying families may have had increased breast awareness and sought early diagnosis (lead-time bias). In the nineteen-nineties several of the mutation carriers had been enrolled into surveillance programs which could also contribute to lead-time bias. Use of new chemotherapy may have increased survival in the controls during the study period. The cases contracted both contralateral cancers and ovarian cancers, all expressions of the same underlying genetic defect. Censoring out other expressions of the same genetic defect, is, however, informative censoring if the question is prognosis in a *BRCA1 *mutation carrier (which may not be identical to prognosis of the first tumour detected). In short, the results were indicative of multiple determining factors which again may explain the fact that different studies have arrived at different conclusions. With respect to different results in other reports, variations in population structures may also have been instrumental.

Considering stage 1 tumours separately, the picture was simpler: The crossing of the curves almost disappeared and a difference between cases and controls became visible (Figure 4)

Comparing the present results with the survival following early diagnosis and treatment of inherited breast cancer [24]; both similarities and differences are obvious: The survival of *BRCA1*-associated breast cancer seems close to identical in the retrospective and prospective series – meaning that the effect of early diagnosis and treatment may have been negligible. In contrast, the other groups in the prospective series (familial breast cancer without demonstrable mutations and BRCA2 mutation carriers) do well – and there is no initial crossing of the curves. The latter observation strengthens the theory that methodological problems in the present retrospective series may be associated with the crossing of the curves.

Whatever interpretation one may choose, the findings were that stage 1 breast cancer in *BRCA1 *carriers has a poor prognosis. These findings are supported by Rennert et al [27] who found that the outcome was worse for BRCA1 mutation carriers with small node-negative tumours. This is worrisome, because it is adds to the reasons for questioning the effect of our programme for early detection and treatment to cure inherited breast cancer [20,21,24], On the other hand, the suggestion of prophylactic mastectomy in young ages – without having proof that it is necessary – is problematic as well. It might be wise to see prospective data on the effect of early diagnosis of breast MRI with respect to survival, before we arrive at firm conclusions and advise all mutation carriers to choose prophylactic mastectomy.

We undertook the present study because the problems discussed are serious and unsolved. We did arrive at the two main conclusions we had expected: The confusion apparent in the litterature may be explained by the fact that the data are confusing. There may be nothing essentially wrong with the results of the present study or any of the previous conflicting reports. The most probable explanation for the diversity of results is that there are several factors with varying influence on the combined results.

The main conclusions in our study, are that *BRCA1 *associated breast cancer has a serious prognosis, and this is especially so for early stages. It questions the current belief that early diagnosis will significantly improve survival in *BRCA1 *mutation carriers.

## Competing interests

The authors declare that they have no competing interests.

## Authors' contributions

AIH conceived of the study, participated in its design, was the data manager, performed the statistical analysis and drafted the manuscript. ST participated in its design and statistical analysis, and provided the control group. JA participated in its design, provided background information and supervised the activities for Haukeland University Hospital. LM participated in its design and coordination and provided background information. NV generated the data and coordinated the study at Radiumhospitalet. PM initiated the study, participated in its design, controlled the statistical analysis and drafted the manuscript. All authors read and approved the final manuscript.
